# Two P_1B-1_-ATPases of *Amanita strobiliformis* With Distinct Properties in Cu/Ag Transport

**DOI:** 10.3389/fmicb.2018.00747

**Published:** 2018-04-23

**Authors:** Vojtěch Beneš, Tereza Leonhardt, Jan Sácký, Pavel Kotrba

**Affiliations:** Department of Biochemistry and Microbiology, University of Chemistry and Technology Prague, Prague, Czechia

**Keywords:** ectomycorrhizal fungi, P_1_-type ATPase, copper transporter, silver transporter, metal homeostasis, *Amanita strobiliformis*

## Abstract

As we have shown previously, the Cu and Ag concentrations in the sporocarps of Ag-hyperaccumulating *Amanita strobiliformis* are correlated, and both metals share the same uptake system and are sequestered by the same metallothioneins intracellularly. To further improve our knowledge of the Cu and Ag handling in *A. strobiliformis* cells, we searched its transcriptome for the P_1B-1_-ATPases, recognizing Cu^+^ and Ag^+^ for transport. We identified transcripts encoding 1097-amino acid (AA) AsCRD1 and 978-AA AsCCC2, which were further subjected to functional studies in metal sensitive *Saccharomyces cerevisiae*. The expression of As*CRD1* conferred highly increased Cu and Ag tolerance to metal sensitive yeasts in which the functional AsCRD1:GFP (green fluorescent protein) fusion localized exclusively to the tonoplast, indicating that the AsCRD1-mediated Cu and Ag tolerance was a result of vacuolar sequestration of the metals. Increased accumulation of As*CRD1* transcripts observed in *A. strobiliformis* mycelium upon the treatments with Cu and Ag (8.7- and 4.5-fold in the presence of 5 μM metal, respectively) supported the notion that AsCRD1 can be involved in protection of the *A. strobiliformis* cells against the toxicity of both metals. Neither Cu nor Ag affected the levels of As*CCC2* transcripts. Heterologous expression of As*CCC2* in mutant yeasts did not contribute to Cu tolerance, but complemented the mutant genotype of the *S. cerevisiae ccc2*Δ strain. Consistent with the role of the yeast Ccc2 in the trafficking of Cu from cytoplasm to nascent proteins via post-Golgi, the GFP fluorescence in As*CCC2*-expressing *ccc2*Δ yeasts localized among Golgi-like punctate foci within the cells. The As*CRD1*- and As*CCC2*-associated phenotypes were lost in yeasts expressing mutant transporter variants in which a conserved phosphorylation/dephosphorylation site was altered. Altogether, the data support the roles of AsCRD1 and AsCCC2 as genuine P_1B-1_-ATPases, and indicate their important functions in the removal of toxic excess of Cu and Ag from the cytoplasm and charging the endomembrane system with Cu, respectively.

## Introduction

Studies have revealed that ectomycorrhizal (EM) fungi effectively mobilize heavy metals from soils and minerals (Gadd et al., 2012) and that ectomycorrhizae improve plant fitness in metal polluted environments also because metal tolerant mycobionts function as a barrier for the entry of metals into plant tissues (Colpaert et al., 2011; Reddy et al., 2016). High concentrations of heavy metals and metalloids accumulated in the sporocarps further support the notion that EM fungi substantially contribute to the environmental cycling of these elements, including Cu and Ag (Falandysz and Borovička, 2013). It is noteworthy that studies indicate that macrofungi could be considered the most effective Ag accumulators among eukaryotes with two known outstanding EM species, *Amanita* *strobiliformis* and *Amanita solitaria* (Borovička et al., 2007, 2010). The concentrations of Ag in their sporocarps collected from unpolluted sites range from 200 to 1200 mg kg^-1^. We have documented that the intracellular detoxification of Cu and Ag in *A. strobiliformis* largely relies upon binding with cysteinyl-rich, cytosolic metallothionein (MT) peptides, AsMT1a, 1b, and 1c (Osobová et al., 2011; Beneš et al., 2016; Hložková et al., 2016); and that two *A. strobiliformis* transporters of the copper transporter family (CTR; specifically AsCTR2 and AsCTR3) can recognize not only Cu, but also Ag for uptake (Beneš et al., 2016).

Studies in eukaryotes have revealed that while CTRs transport Cu ions into the cytoplasm, the members of P_1B-1_ subgroup of P_1B_-type ATPases (also called heavy metal ATPases, HMA) contribute to the homeostasis and redistribution of essential Cu by exporting the metal ion from the cytoplasm into the subcellular compartments or out of the cell (Nevitt et al., 2012; Bashir et al., 2016). The homology of P_1B_-ATPases and their characteristic sequence features suggest a division into seven subgroups (Smith et al., 2014). While the roles of the members of the P_1B-5_ to P_1B-7_ subgroups (predicted so far only in prokaryotes) remain elusive, the transporters belonging to P_1B-1_, P_1B-2_, prokaryote P_1B-3_, and P_1B-4_ subgroups are known for distinct preferences for their substrate heavy metal ion(s). The transporters highly specific for monovalent Cu ions (the dominant intracellular Cu species in eukaryotes; Nevitt et al., 2012) comprise P_1B-1_-subgroup, while P_1B-2_, P_1B-3_, and P_1B-4_ transport Cd^2+^/Zn^2+^/Pb^2+^, Cu^+^/Cu^2+^, and Co^2+^, respectively.

The intracellular handling of Cu involves in *Saccharomyces cerevisiae* Ccc2 protein (Bleackley and MacGillivray, 2011), and in mammals the Menkes protein ATP7A and Wilson protein ATP7B (La Fontaine and Mercer, 2007; Nevitt et al., 2012). These P_1B-1_-ATPases are responsible for the transport of the physiological Cu into the post-Golgi. Unlike with Ccc2 in *S. cerevisiae*, the Cu overload in mammalian cells triggers trafficking of ATP7A to the plasma membrane and ATP7B to the excretory vesicles, and both transporters then facilitate the efflux of the excess metal to rescue the cell from Cu toxicity. Similar trafficking [from the endoplasmic reticulum (ER) to the plasma membrane] stimulated by Cu overload has been documented in *Arabidopsis thaliana* for its AtHMA5 and heterologously expressed SvHMA5I from *Silene vulgaris* (Li et al., 2017). It is noteworthy that several P_1B-1_-ATPases have been shown to also recognize Ag for transport (Argüello et al., 2007; Smith et al., 2014; Migocka et al., 2015). Among fungi, the plasma membrane Cu^+^- and Ag^+^-efflux CaCRD1 of *Candida albicans* provides the primary source of cellular resistance against both metals (Riggle and Kumamoto, 2000; Weissman et al., 2000). Recently, the P_1B-1_-ATPase CrpA that also localizes to the plasma membrane has been shown to confer substantial Cu- but not Ag-tolerance in filamentous fungus *Aspergillus nidulans* (Antsotegi-Uskola et al., 2017).

Since our previous studies revealed certain overlap in the cell biology of Ag and Cu in *A. strobiliformis*, we investigated whether or not this species may employ P_1B-1_-ATPases in the intracellular handling of both Cu and Ag. We searched its transcriptome for the homologs of P_1B-1_-ATPases and describe here the isolation and functional characterization of cDNA coding the Cu- and Ag-inducible AsCRD1 that can protect metal-sensitive yeasts against the toxicity of both metals. We also describe the second isolated P_1B-1_-ATPase of *A. strobiliformis*, the homolog of yeast Ccc2 named AsCCC2. To our knowledge, these are the first P_1B-1_-ATPases characterized in mycorrhizal fungi.

## Materials and Methods

### Amplification of As*CRD1* and As*CCC2* Genes and Sequence Analyses

Partial sequences of As*CRD1* and As*CCC2* transcripts were obtained from tBLASTn analysis (Altschul et al., 1990) of the transcriptome of *A. strobiliformis* (Paulet ex Vittad.) isolate PRM 857486 (Hložková et al., 2016) by using characterized fungal P_1B-1_-type ATPases as queries. The entire coding sequence information was established by 5′ and 3′ RACE, using a SMARTer RACE cDNA Amplification Kit (Clontech Labs) with 1 μg of total RNA to produce the population of the first cDNA strand; the Q5 High-Fidelity DNA polymerase (New England Biolabs) was used to obtain double-stranded cDNAs. The total RNA was isolated by using an RNeasy Plant Mini Kit and RNase free DNase set (Qiagen) from 50 mg of freeze-dried tissue of the *A. strobiliformis* PRM 857486 sporocarp. Transcript-specific primers were 5rCRD1_R1 to R5 for As*CRD1* 5′ RACE, and 5rCRD2_R1 to R3 or 3rCRD2R1 and R2 for As*CCC2* 5′ or 3′ RACE, respectively (for primer sequences see **Supplementary Table S1**), and the amplicons were subjected to 3′-A tailing with GoTaq DNA polymerase (Promega). Genomic fragments harboring As*CRD1* and As*CCC2* genes were amplified from 200 ng of chromosomal DNA template by PCR using Q5 DNA polymerase and pairs of gene-specific primers designed based on 5′ and 3′ untranslated regions of the corresponding cDNAs; the primers were CRD1_F/R for As*CRD1* and CRD2_F/R for As*CCC2* (**Supplementary Table S1**). The chromosomal DNA was isolated from 50 mg of freeze-dried tissue of *A. strobiliformis* PRM 857486 by using a NucleoSpin Plant II Kit (Macherey-Nagel). The amplicons were inserted to a pGEM-T vector (Promega) and then amplified in *E. coli* DH5α according to standard protocols. The recombinant DNAs were subjected to custom DNA sequencing on both strands with the vector-specific primers. The sequences of As*CRD1* and As*CCC2* cDNAs were deposited in GenBank under the accession numbers MF317930 and MF317931, respectively.

### Sequence Analyses

The protein sequences deduced from the cDNAs were subjected to a transmembrane domain and signal peptide predictions *in silico* at the CCTOP web server (Dobson et al., 2015). The signal peptide prediction was also done by submitting the sequences to SignalP 4.1 server (Pettersen et al., 2004). The homology modeling of transporter 3D structure used the Phyre2 protein homology/analogy recognition engine (Kelley et al., 2015), the Modeller (Webb and Sali, 2014), and UCSF Chimera (Pettersen et al., 2004) programs. The closest AsCRD1 and AsCCC2 homologs among the RCSB Protein Data Bank (PDB) entries used for comparative modeling were 2EW9 (N-terminal domain of ATP7B, 23% and 40% identity, respectively) and 3J09 (P_1B-1_-ATPase of *Archaeoglobus fulgidus*; 34% and 41% identity, respectively). A MEGA 6.0 package (Tamura et al., 2013) incorporating ClustalW (Thompson et al., 1994) was used to align AsCRD1, AsCCC2, and related amino acid (AA) sequences (retrieved from UniProtKB datase by using BLASTp) and construct the corresponding unrooted phylogenetic tree using the Neighbor-joining method with Poisson correction model and 10,000 bootstrap replications.

### Functional Complementation in Yeasts

The *S. cerevisiae* strains used in complementation assays were *cup1*Δ strain DTY113 (*MATα trp1-1 leu2-3,-112 gal1 ura3-50 cup1*Δ*61*; Tamai et al., 1993) and the Euroscarf^1^ Y00569 (*yap1*Δ; YML007w::kanMX4) and Y03629 (*ccc2*Δ; YDR270w::kanMX4) mutant strains of BY4741 (*MAT*a *his3Δ1 leu2Δ0 met15Δ0 ura3Δ0*). To constitutively express As*CRD1* and As*CCC2* in yeasts, the entire coding sequences produced by Q5 DNA polymerase from cDNA using primer pairs eifCRD1_F/R (As*CRD1*) and eifCRD2_F/R (As*CCC2*) were inserted into the HindIII-treated and EcoRI-treated yeast expression vector p416GPD (Mumberg et al., 1995), respectively, by using an In-Fusion HD Cloning Kit (Clontech Labs) according to manufacturer’s instructions. Site-directed mutagenesis of As*CRD1* and As*CCC2* in p416GPD was performed by the inverse PCR method (Füzik et al., 2014) with Phusion High-Fidelity DNA Polymerase (Thermo Scientific); the overlapping primers used were mCRD1_F plus mCRD1_R and mCRD2_F plus mCRD2_R, respectively. Primer sequences are listed in **Supplementary Table S1**. The yeasts transformed with p416GPD-based plasmids were routinely grown at 30°C on *URA*^-^ selective SD medium containing (w/v) 0.7% yeast nitrogen base (Difco), 0.005% adenine hemisulfate, 2% glucose, and 0.003% of each of the essential amino acids (Sigma-Aldrich).

For complementation plate assays, the mid-log cultures of transformed *S. cerevisiae* were adjusted to an optical density at 590 nm (OD_590_) of 0.1, and 5 μl of serial dilutions were spotted on agar medium. The metal (added as CuCl_2_ or AgNO_3_) tolerance of *cup1*Δ and *yap1*Δ transformants was assayed on SD medium and non-fermentable YPEG medium [1% (w/v) yeast extract, 2% ([w/v) peptone, 2.5% (v/v) ethanol and 2.5% (v/v) glycerol], respectively. The growth tests of *ccc2*Δ transformants used non-fermentable YPEG medium.

### Fluorescence Microscopy of As*CRD1:GFP* and As*CCC2:GFP*-Expressing Yeasts

To construct the translational As*CRD1*:*GFP* and As*CCC2*:*GFP* fusions, the coding sequences without the termination codons were amplified from cDNA by using primer pairs gifCRD1_F plus gifCRD2_R for As*CRD1* and gifCRD2_F plus gifCRD2_R for As*CCC2* (**Supplementary Table S1**). The amplicons were inserted into a BamHI-digested plasmid p416GFP. The plasmid p416GFP is a p416GPD derivative, harboring *GFP* from plasmid pEGFP-C1 (Clontech Labs) inserted as a BamHI/HindIII DNA fragment (Hložková et al., 2016). The cells of As*CRD1*:*GFP*-expressing *cup1*Δ and As*CCC2*:*GFP*-expressing *ccc2*Δ yeasts were obtained from mid-log cultures grown in SD medium supplemented with 0.5 μg⋅ml^-1^ DAPI (Invitrogen) when needed. Vacuoles were labeled at 30°C for 4 h in SD medium with 400 μg⋅ml^-1^ of the tonoplast-specific FM4-64 dye (Molecular Probes). The fluorescence microscopy was performed by using a BioSystems Imaging station CellˆR with a MT20 illumination and a DSU semiconfocal unit on a IX-81 microscope (Olympus BioSystems) equipped with the model C9100 EM-CCD camera (Hamamatsu Photonix). A GFP-deriving fluorescence was observed with the U-DM-DA-FI-Tx2 FITC filter (excitation band: 495/15 nm, emission band: 530/30 nm; Olympus) and nuclei stained with DAPI were visualized with the U-DM-DA-FI-Tx2 DAPI filter (excitation band: 400/15 nm, emission band: 460/20 nm). Vacuoles were observed with the U-DM-Cy5 filter (excitation band: 590–650 nm, emission band: 665–740 nm). The recorded black and white images were processed using the ImageJ software^2^.

### Gene Expression Analysis in *A. strobiliformis*

The mycelium isolate from the PRM 857486 pileus (Osobová et al., 2011) was grown at 25°C and routinely maintained on potato dextrose (PD) agar containing 4 g⋅l^-1^ potato extract (Sigma-Aldrich) and 10 g⋅l^-1^ glucose (0.5× PD). The metal dose-dependent growth was observed with mycelia grown for 8 weeks on 0.5× PD agar with CuCl_2_ or AgNO_3_ supplements. The expression of target genes was assessed in the mycelium propagated in liquid PD medium (basal Cu, Ag and Cd concentrations below the atomic absorption spectrometry detection limit of 0.21, 0.04, and 0.09 μM, respectively) for 16 weeks and then subjected to metal (added as CuCl_2_, AgNO_3_, or CdCl_2_) exposures for 24 h. The gene expression analysis was performed on independent biological samples from three replicate experiments in two technical replicates. The RNA extraction from freeze-dried mycelia and quantitative reverse-transcribed PCR measurements including the quality/specificity controls were conducted essentially as described previously (Hložková et al., 2016). Briefly, the population of transcripts present in 1 μg of total RNA was reverse transcribed in a 20 μl reaction and 1.5 μl of the resulting cDNA product was used in a 12 μl quantitative PCR (qPCR) reaction for the measurements with 0.35 μM gene-specific primers (**Supplementary Table S1**). The measurements used a DyNAmo Flash SYBR Green 2-Step qPCR Kit (Life Technologies) and a MiniOpticon Real Time PCR System (Bio-Rad). The primers were qF- plus qR-CRD1 for As*CRD1*, qF- plus qR-CRD2 for As*CRD1*, and qFtub-b plus qRtub-b for β-tubulin As*TUB-b* gene (GenBank: JX463743), which was used for normalization of the qPCR data as internal reference, stably expressed under Ag and Cu exposures (Hložková et al., 2016). A Bio-Rad CFX Manager was used to calculate the baseline range and the experiment threshold cycle (C_te_) values recorded during the elongation period of the qPCR. The levels of gene transcription as relative to the controls (unexposed mycelium) were calculated by using the 2^-ΔΔCt1^ method (Livak and Schmittgen, 2001), where C_t1_ = C_te_ × [log(1+*E*)/log2]. The amplification efficiency values *(E)* were calculated using the equation *E =* [10^(-1/slope)^]-1; the slopes were determined from the standard quantification curves obtained with serial dilutions of first strand cDNA templates. The obtained *E* values for As*CRD1*, As*CCC2* and As*TUBb* genes were 102%, 98%, and 108%, respectively.

## Results

### Identification and Sequence Analysis of AsCRD1 and AsCCC2

To obtain information about the sequences coding for P_1B-1_-ATPases in *A. strobiliformis*, the sporocarp transcriptome of *A. strobiliformis* was screened by using tBLASTn search with known P_1B-1_-ATPases as queries. The screening retrieved two partial transcript sequences: one 822 nucleotides long in which a termination codon was included (a part of mRNA named As*CRD1*) and another 528 nucleotides long without a termination codon (a part of mRNA named As*CCC2*). As the deduced protein fragments showed a substantial identity with the C-terminal sequences of known P_1B-1_-ATPases, the corresponding full-length coding sequences were established via the RACE method.

**FIGURE 1:**
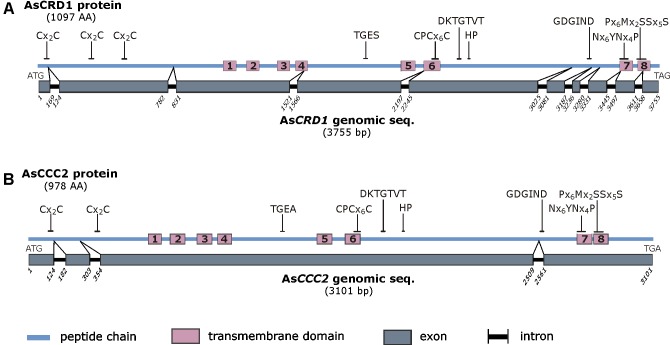
Schematic representation of the predicted AsCRD1 protein **(A)** and AsCCC2 protein **(B)** and the corresponding genomic sequences. The characteristic amino acid sequence motifs are indicated and include Cx_2_C metal binding motif, sequence features characteristic of phosphatase domain (TGE[S/A]), aspartyl kinase domain (DKTGTxT), HP locus (HP), ATP-binding domain (GDGxND), Cu translocation domain (CPCx_5_P), and P_1B-1_-subtype signature sequences (Nx_6_YNx_4_P and Px_6_MxxSSx_5_S). For the comparative amino acid sequence analysis and homology models see **Supplementary Figure S1**.

The predicted 1097-AA AsCRD1 and 978-AA AsCCC2 proteins showed the characteristic sequence features of P_1B-1_-ATPases described in other organisms (Argüello et al., 2007; Smith et al., 2014). These involve putative N-terminal Cu/Ag-binding CxxC motifs (three in AsCRD1, two in AsCCC2) and two P_1B-1_ subgroup signature sequences in predicted transmembrane domains (TMD), Nx_6_YNx_4_P (x represents any AA residue), and Px_6_MxxSSx_5_S, which are in P_1B-1_-ATPases conserved in TMD7 and TMD8, respectively (**Figure 1** and **Supplementary Figure S1**). Like other P_1B_-type ATPases, AsCRD1 and AsCCC2 contained eight predicted TMDs with CPCx_6_P sequence in TMD6 and HP locus between TMD6 and TMD7. In addition, both predicted proteins possess features typical for all the members of the P-ATPase superfamily (**Figure 1**), particularly the DKTGTxT motif in the predicted large cytoplasmic loop with an aspartyl residue whose phosphorylation from ATP and dephosphorylation is prerequisite for active metal ion transport (Palmgren and Nissen, 2011). Despite the identified regions of conservancy at the protein level, the corresponding genes showed different structure and appeared dissimilar. The cDNA and genomic sequences of As*CRD1* and As*CCC2* were clearly distinct, with coding sequences interrupted with nine and three introns, respectively (**Figure 1**).

The comparison of the predicted AsCRD1 and AsCCC2 proteins revealed that along the sequence, they show lower identity and similarity with each other (25% and 38%, respectively) than they individually showed to P_1B-1_-ATPases characterized from other species. Predicted AsCRD1 shares 38%, 36%, and 31% identity (54%, 50%, and 48% similarity) with *A. nidulans* CrpA, *C. albicans* CaCRD1, and cucumber (*Cucumis sativus*) CsHMA5.2, respectively, while AsCCC2 shows 35% identity and 51% similarity with both the *S. cerevisiae* Ccc2 and *A. thaliana* AtHMA5. As further indicated in the Neighbor-joining tree (**Supplementary Figure S2**), AsCRD1 and AsCCC2 sort into two distinct clusters. The AsCRD1-containing cluster comprised the characterized CaCRD1 and a clade of predicted agaricomycete P_1B-1_-ATPases. The second cluster involved clearly separated clades of mammalian and plant P_1B-1_-ATPases together with the AsCCC2-containing agaricomycete clade, which was more closely related to plant than to mammalian or yeast transporters. It is noteworthy that among the characterized transporters from Ascomycetes and Basidiomycetes, the closest relatives of AsCCC2 were P_1B-1_-ATPases from plant pathogens *Botrytis cinerea* (Saitoh et al., 2010) and *Colletotrichum lindemuthianum* (Parisot et al., 2002), and human pathogen *Cryptococcus neoformans* (Walton et al., 2005).

### Functional Expression of AsCRD1 and AsCCC2 in *S. cerevisiae*

The homology to known fungal P_1B-1_-ATPases suggested that AsCRD1 and AsCCC2 are P_1B-1_-ATPases, which could be involved in metal tolerance and delivery of Cu to metalloproteins, respectively. In order to gain information regarding the function of AsCRD1 and AsCCC2 in handling Cu and Ag, the corresponding coding sequences were constitutively expressed in mutant *S. cerevisiae* strains grown on agar media with or without metal supplements. To attest the importance of the DKTGTxT motif in which the conserved aspartyl is in P-ATPases, a target of phosphorylation/dephosphorylation during the transport reaction cycle, the corresponding mutant As*CRD1*^D742A^ and As*CCC2*^D555A^ variants were constructed, in which the codons for aspartyl 742 (in As*CRD1*) and aspartyl 555 (in As*CCC2*) were changed to encode alanyl residues.

**FIGURE 2:**
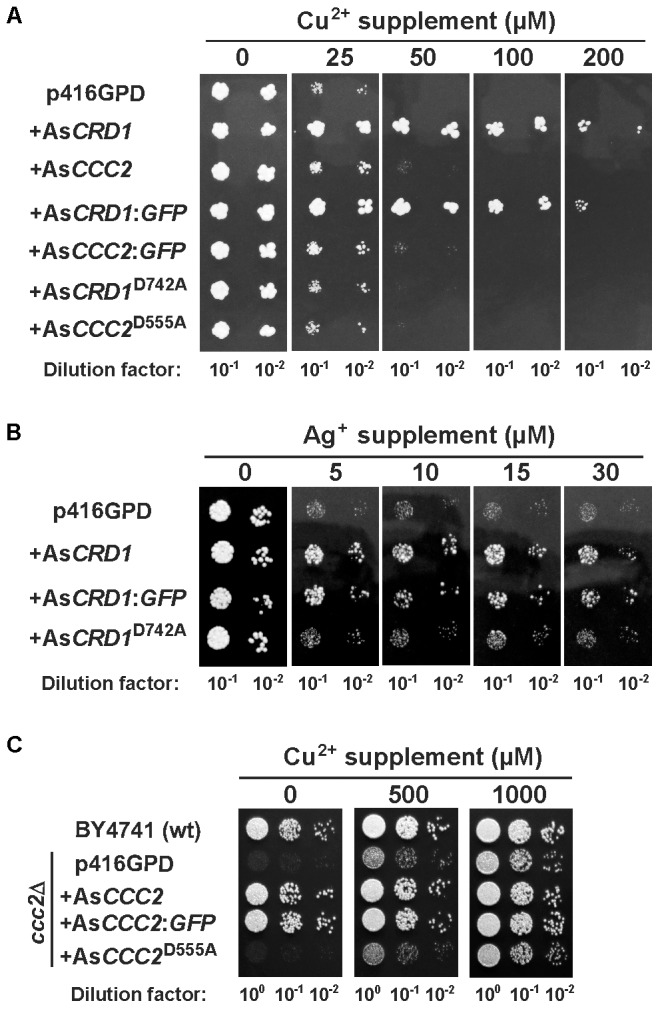
Metal-related phenotypes of yeasts expressing As*CRD1* and As*CCC2* variants. **(A)** Cu tolerance and **(B)** Ag tolerance of the indicated transformants of Cu-hypersensitive *cup1*Δ and Ag-sensitive *yap1*Δ strains of *S. cerevisiae*, respectively. **(C)** Growth of the indicated transformants of *ccc2*Δ strain under respiratory conditions. Spotted for growth were the diluted cultures of cells transformed with the empty p416GPD vector or with the same expression vector inserted with As*CRD1* or As*CCC2*, their translational fusions with GFP, or mutant variants (As*CRD1*^D742A^, As*CCC2*^D555A^). Metal tolerance assays were performed using SD medium with or without indicated metal supplement and assays with *ccc2*Δ strain were conducted on non-fermentable YPEG medium.

The Cu tolerance assays were conducted in the *cup1*Δ strain carrying a deletion of its single-copy MT gene *cup1*, which renders the cells hypersensitive to Cu. Heterologous expression in yeasts grown on SD medium containing 50 or 100 μM Cu^2+^ revealed that only As*CRD1*, but not As*CCC2*, protected the yeasts form Cu toxicity (**Figure 2A**). The protective effect of As*CRD1* became weaker when the cells were subjected to 200 μM Cu^2+^ (**Figure 2A**). Considering that Ag^+^, particularly in respiratory conditions, acts as a potent inducer of oxidative stress (Mijnendonckx et al., 2013), and yeasts with defects in oxidative stress response proved useful in attributing Ag-detoxification functions to heterologous proteins (Sácký et al., 2014; Migocka et al., 2015), the *yap1*Δ strain, deficient in a transcription factor upregulating genes involved in oxidative stress response (Rodrigues-Pousada et al., 2010), was used in Ag toxicity assays. As documented in **Figure 2B**, the *yap1*Δ cells grown on non-fermentable YPEG medium and expressing As*CRD1* grew much better in the presence of 5–30 μM Ag^+^ than did the controls. The observation that the expression of As*CRD1*^D742A^ did not confer increased resistance against either Cu (**Figure 2A**) or Ag (**Figure 2B**) suggested that the Cu- and Ag-tolerance phenotypes associated in the model yeasts with wild-type As*CRD1* were indeed due to the metal-transport ability of the encoded protein.

The apparent lack of the Ag/Cu toxicity-related phenotype of As*CCC2* in *cup1*Δ and *yap1*Δ yeasts was congruent with the expected function of AsCCC2 as the transporter involved in handling of physiological Cu inside the cell. The properties of AsCCC2 were thus further tested in the *ccc2*Δ strain in which the absence of Ccc2 causes a severe growth defect on non-fermentable media because of the lack of sufficient mitochondrial iron (Fu et al., 1995; Yuan et al., 1997); note that high affinity iron uptake pathway in *S. cerevisiae* involves Fet1 permease that works together with Cu-dependent, plasma membrane ferroxidase Fet3 that receives its Cu ions (supplied by Ccc2) in Golgi. The growth tests on YPEG medium revealed that As*CCC2* was able to fully complement the respiratory deficiency of the *ccc2*Δ cells, whilst the control cells transformed with empty p416GPD and those expressing As*CCC2*^D555A^ (and As*CRD1*; not shown) failed to grow under the same conditions (**Figure 2C**). The controls, As*CRD1* (not shown), and As*CCC2*^D555A^ cells showed full growth on the YPEG medium supplemented with 1 mM Cu^2+^, respectively.

### Targeting of AsCRD1 and AsCCC2 in *S. cerevisiae*

Distinct phenotypes associated with As*CRD1* and As*CCC2* in yeasts suggested that the corresponding proteins localized to different membranes. To obtain information about the cellular localization of AsCRD1 and AsCCC2 using direct fluorescence microscopy, the proteins were translationally fused with GFP at their C-termini, and the recombinant As*CRD1*:*GFP* and As*CCC2*:*GFP* genes were expressed in *cup1*Δ and *ccc2*Δ yeasts grown in SD medium. Complementation assays revealed that the phenotypes conferred by the fusions upon the yeasts were essentially the same as those observed with the corresponding transporters without GFP (**Figure 2**), thereby indicating that AsCRD1 and AsCCC2 tagged with GFP at their C-termini remained functional.

**FIGURE 3:**
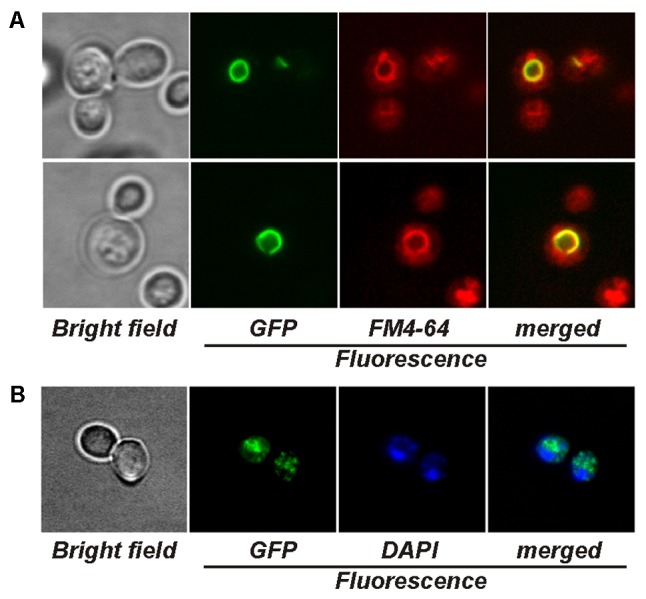
Visualization of yeasts producing GFP-tagged AsCRD1 and AsCCC2 by fluorescence microscopy. **(A)** Cells of *cup1*Δ strain expressing As*CRD1*:*GFP*. The vacuoles were stained with FM4-64; displayed panels from left to right: green GFP fluorescence, red FM4-64 fluorescence, and GFP/FM4-64 merged image with yellow GFP and FM4-64 overlap. **(B)** Cells of *ccc2*Δ strain expressing As*CCC2*:*GFP*. The nuclei were stained with DAPI; displayed panels from left to right: green GFP fluorescence, blue DAPI fluorescence, and GFP/DAPI merged image.

The microscopy of As*CRD1*:*GFP*-expressing *cup1*Δ yeasts revealed strong GFP fluorescence co-localizing exclusively with the tonoplast stained with the vacuole-specific fluorophore FM4-64 (**Figure 3A**). The expression of As*CCC2*:*GFP* in the *ccc2*Δ strain resulted in a strong, punctuated GFP signal in vesicular bodies within the cell (**Figure 3B**). The absence of GFP fluorescence from the perinuclear region attributable to ER may suggest that AsCCC2:GFP localized to Golgi rather than ER. The localization of GFP fluorescence in As*CRD1*:*GFP-* and As*CCC2*:*GFP-*transformed yeasts was not affected by the presence of subtoxic concentrations of Cu or Ag or the length of culture period (not shown).

### Metal Responsiveness of As*CRD1* and As*CCC2* in *A. strobiliformis*

**FIGURE 4:**
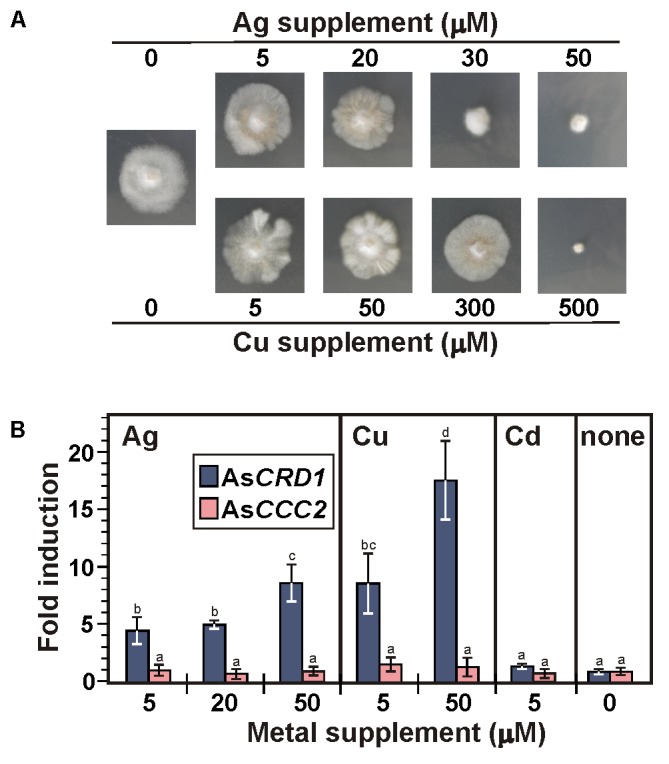
Growth of *A. strobiliformis* and expression of As*CRD1* and As*CCC2* genes in the presence of metal ions as indicated. **(A)** Mycelia grown on 0.5× PD medium with or without metal supplement for 8 weeks (long-term exposure). **(B)** Relative transcript levels measured by qRT-PCR in mycelium incubated in liquid 0.5× PD medium for 24 h with or without (the controls) metal supplement (short term exposure). Expression of β-tubulin gene was used to calculate the relative expression and values plotted are an average of three biological replicates ± standard deviation of the mean (different letters above the bars indicate significant differences as determined by ANOVA followed by Tukey’s test, *p* < 0.05).

Considering the As*CRD1*-associated, metal tolerance-related phenotypes in the model yeasts and the typically induced expression of metal tolerance genes during metal overload, the transcription rates of the studied P_1B-1_-ATPases genes were analyzed by using qRT-PCR, measuring mRNA levels in the mycelium of *A. strobiliformis* treated with 5 and 50 μM Cu^2+^, 5, 20, and 50 μM Ag^+^, or 5 μM Cd^2+^ for 24 h. The Cu and Ag concentrations used in the 24-h exposures proved sublethal also in long-term exposures (**Figure 4A**), although the radial growth of mycelia was strongly reduced (by 70%) in the presence of 50 μM Ag. The mycelia always developed brown zones already at 5 μM of any of the metals, presumably due to the induced production of stress-related melanin (Gostinčar et al., 2012).

As shown in **Figure 4B**, 24 h treatments of mycelia with Ag and Cu clearly elevated the expression of As*CRD1*, but not As*CCC2*, relative to the unexposed control. The average levels of As*CRD1* transcripts increased 4.5- and 8.7-fold in the presence of 5 μM Ag^+^ and Cu^2+^, respectively, and they further nearly doubled when the concentration of the two metals was 50 μM. Neither As*CRD1* nor As*CCC2* showed significant response when the mycelia were treated with a 5 μM concentration of Cd^2+^ (**Figure 4B**), which in *A. strobiliformis* induces the expression of Zn^2+^/Cd^2+^-related MT gene As*MT3*, but not Cu^+^/Ag^+^-related As*MT1*s (Hložková et al., 2016).

## Discussion

Our previous studies revealed a certain overlap in the cellular biology of Cu and Ag in the EM, Ag-hyperaccumulating fungus *A. strobiliformis* – both metals can enter the cells via AsCTR2 and AsCTR3 transporters (Beneš et al., 2016) and intracellular Cu and Ag are sequestered in the cytoplasm through binding with AsMT1s (Hložková et al., 2016). It is worth noting that MTs have been considered principal in the sequestration of Cu or Ag in many EM fungi, including *Pisolithus albus* (Reddy et al., 2016), *Laccaria bicolor* (Reddy et al., 2014), *Hebeloma mesophaeum* (Sácký et al., 2014), *Hebeloma cylindrosporum* (Ramesh et al., 2009), *Amanita submembranacea* (Borovička et al., 2010), and *Paxillus involutus* (Bellion et al., 2007). The present study aimed to identify P_1B-1_-ATPases of *A. strobiliformis* and inspect their potential role in the handling of intracellular Cu and Ag in this species. Our search of the sporocarp transcriptome suggested the presence of several putative P_1B_-ATPases of which only two showed sequence features characteristic of the P_1B-1_ subgroup.

Unlike for Zn or Cd, information about the deposition of the excess of the accumulated Cu in fungal vacuoles is scarce. In *Aspergillus niger* (Fomina et al., 2007) and in arbuscular mycorrhizal *Rhizophagus intraradices* (González-Guerrero et al., 2008), the vacuolar sequestration of excess Cu was revealed by X-ray microanalyses, which further suggested the association of Cu with vacuolar polyphosphate in *A. niger*. The vacuole is an important organelle for Cu homeostasis in *S. cerevisiae* and the strains defective in vacuolar assembly are hypersensitive to Cu (Szczypka et al., 1997). While the transporters of the CTR family responsible for the mobilization of the vacuolar Cu back into the fungal cytoplasm are well characterized (e.g., Ctr2 in *S. cerevisiae*; Bleackley and MacGillivray, 2011), the Cu-specific, high-affinity transporters that can deliver Cu into the vacuoles remained elusive.

Besides the sequence features common in P-ATPases, in particular of the P_1B_-subtype, several lines of experimental evidence implicate that AsCRD1 can act as a detoxification P_1B-1_-ATPase that can transport Cu^+^ and Ag^+^ into vacuoles in *A. strobiliformis*. First, the expression of As*CRD1*, but not As*CRD1*^D742A^, protected the model yeasts from Cu and Ag toxicity. The observation that the replacement of aspartyl with alanyl in the DKTGTxT motif (to prevent the phosphorylation in AsCRD1^D742A^ from ATP) abolished the As*CRD1*-associated phenotype in both *cup1*Δ and *yap1*Δ yeast mutants further indicates that AsCRD1 can recognize both Cu and Ag for an active, ATP-dependent transport, and that it was the metal transport that increased the metal tolerance in the yeasts, not a mere immobilization of Cu^+^ or Ag^+^ through binding to cytoplasmic N-terminal metal binding motifs as it is the case, e.g., of Cu-binding to the Cd-transporting PCA1 in *S. cerevisiae* (Adle et al., 2007). Second, the functional GFP-tagged AsCRD1 was targeted to the tonoplast in model yeasts. Although vacuolar P_1B-1_-ATPases have not been described in fungi before, such localization is not without precedent. Recent studies in plants have identified the Cu-transporting *S. vulgaris* SvHMA5II (Li et al., 2017), and cucumber (*Cucumis sativus*) Cu- and also Ag-activated CsHMA5.1 and CsHMA5.2 proteins (Migocka et al., 2015), as tonoplast-localizing P_1B-1_-ATPases facilitating metal detoxification in root cells. Third, the observation that the expression of As*CRD1* was in *A. strobiliformis* effectively induced by Cu and Ag makes it reasonable to assume that AsCRD1 is involved in the cellular biology of both metals and the fungus raises the levels of As*CRD1* to handle excess intracellular Cu and Ag. Considering that our previous metal speciation analyses using size exclusion chromatography revealed that the majority of the Ag and Cu accumulated in *A. strobiliformis* is stably bound in 6-kDa complexes with Ag- and Cu-inducible, cytosolic AsMT1s (Osobová et al., 2011; Beneš et al., 2016; Hložková et al., 2016), one may then ask the question of what role AsCRD1 would have in metal detoxification. We propose that vacuolar storage could provide the second line of defense against high intracellular Ag and Cu levels, perhaps during a temporal deficiency of Cu^+^- and Ag^+^-binding AsMT1s, akin to the function of zincosome vesicles acting as transient stores of the excess accumulated Zn in *S. cerevisiae* (Devirgiliis et al., 2004). However, considering the plasma membrane localization of the closely related CaCRD1 in *C. albicans* (Riggle and Kumamoto, 2000; Weissman et al., 2000) and CrpA in *A. nidulans* (Antsotegi-Uskola et al., 2017), the possibility that AsCRD1 mislocalizes in *S. cerevisiae* and in *A. strobiliformis* acts as a transporter that exports the excess Cu and Ag out of the cells should not be excluded.

The predicted AsCCC2 and its homologs from Agaricomycetes appeared phylogenetically associated with the Ccc2 protein from the unicellular basidiomycete *C. neoformans* and to a lesser extent with Ccc2s from ascomycetes *B. cinerea, C*. *lindemuthianum* and *S. cerevisiae*. Congruent with this observation, As*CCC2* functionally complemented the *CCC2* gene in *S. cerevisiae ccc2*Δ that is unable to charge its multicopper oxidase Fet3 with Cu in Golgi to establish the Fet3-Ftr1-based iron uptake system (Bleackley and MacGillivray, 2011). The lack of the As*CCC2*-associated phenotype resulting from the D-to-A substitution in the DKTGTxT motif of the encoded protein (in the *ccc2*Δ cells expressing As*CCC2*^D555A^), and the GFP fluorescence localizing to the intracellular punctuate bodies resembling Golgi in yeasts expressing As*CCC2:GFP* provides further support to the notion that AsCCC2 can mediate active transport of Cu into the Golgi. In *C. neoformans, B. cinerea*, and *C. lindemuthianum*, the corresponding functional *CCC2* gene appeared critical for the biosynthesis of melanin; the lack of CCC2 in these species lead to a disruption in the delivery of Cu to extracellular multicopper oxidases(laccases in particular) during their trafficking through Golgi (Parisot et al., 2002; Walton et al., 2005; Saitoh et al., 2010). Multiple copies of laccase genes have been predicted in both saprobic and EM species (Kohler et al., 2015); for example, the genomes of saprobic *Amanita thiersii* and EM *Amanita muscaria* contain 15 and 18 putative non-allelic laccase genes, respectively. Recent studies indicate that laccases expressed in EM fungi are, besides the pigmentation, involved in the sporocarp development or nutrient acquisition in extraradical mycelia (Courty et al., 2009; Kües and Rühl, 2011; Ellström et al., 2015; Shah et al., 2016). Considering this and the fact that Fet3-like ferroxidase genes have been found in most sequenced basidiomycetes, including *Amanita* species (Kües and Rühl, 2011; Kohler et al., 2015), it could be possible that *A. strobiliformis* benefits from AsCCC2 for both the Fe-uptake complex and laccase(s) assembly via Cu handling.

The results obtained in this study indicate that AsCRD1 and AsCCC2 belong to two separate protein clusters of the P_1B-1_-ATPase subgroup. The collected data strongly suggest that AsCRD1 is in *A. strobiliformis*, like AsMT1s and AsCTRs, involved in the handling of both Ag and Cu, specifically in supporting the detoxification of Ag and Cu, which is, besides efficient transport, the prerequisite for (hyper)accumulation. Our data further indicate that AsCCC2, identified as another P_1B-1_-ATPase of *A. strobiliformis*, is a functional homolog of yeast Ccc2, involved in the delivery of physiological Cu into organelles of endomembrane system for the biosynthesis of Cu-dependent proteins. It is worth noting that BLASTp returned putative P_1B-1_-type ATPases of Agaricomycetes species in which homologs of AsCRD1 and AsCCC2 were identified. These species belong to different orders (**Supplementary Figure S2**) of different lifestyles. It is thus tempting to speculate that the functional specialization and roles of P_1B-1_-type ATPases, which we here discussed for AsCRD1 and AsCCC2, are widespread among Agaricomycetes.

## Author Contributions

VB conducted the experimental work and analyzed and interpreted data. TL and JS jointly contributed to the conception and design of the study, the bioinformatic analyses, and helped with the interpretation of data. PK was responsible for the concept and design of the work and the interpretation of the results, ensured the scientific issue was appropriately investigated, and wrote the manuscript. All of the authors assisted in writing the manuscript, discussed the results, and commented on the manuscript.

## Conflict of Interest Statement

The authors declare that the research was conducted in the absence of any commercial or financial relationships that could be construed as a potential conflict of interest.

## Footnotes

1 http://web.uni-frankfurt.de/fb15/mikro/euroscarf/

2 http://imagej.nih.gov/ij/

## Supplementary Material

FIGURE S1 Comparative sequence analysis of the predicted AsCRD1 and AsCCC2 proteins. Sequences were aligned by using ClustalW and the identical, conservative, and semiconservative residues were marked with asterisks, double dots, and single dots, respectively. The predicted transmembrane domains (TMD, numbered) are highlighted with light blue background and the characteristic sequence motifs are boxed in yellow (metal binding CxxC motif), green (CPCx_6_P in TMD6, Nx_6_YNx_4_P in TMD7, Px_6_MxxSSx_5_S in TMD8, and HP locus), and red (DKTGTxT motif). The signal peptide predictions in AsCRD1 and AsCCC2 at CCTOP and Signal P 4.1 servers did not unveil any potential signal sequences. **(B)** 3D homology models of AsCRD1 and AsCCC2. The PDB entries used for the comparative modeling of AsCRD1 and AsCCC2 were 2EW9 (N-terminal domain of ATP7B, 23% and 40% identity, respectively) and 3J09 (P_1B-1_-ATPase CopA of *Archaeoglobus fulgidus*; 34% and 41% identity, respectively). The positions of characteristic sequence motifs are indicated with arrows.

FIGURE S2 An unrooted, neighbor-joining-based tree of characterized and predicted P_1B-1_-ATPases. Species name and UniProt accession numbers of functionally characterized ascomycete, plant and mammalian, and predicted Agaricomycetes P_1B-1_-ATPases (30% to 40% identical with AsCRD1 or AsCCC2; UniProt expect values of 0 to 10^-97^) are indicated. The tree was generated by MEGA version 6.0 after the sequence alignment by using ClustalW. Bootstrap values (%; 1,000 replicates) are shown at nodes (values <40% are omitted for clarity) and branch lengths are proportional to phylogenetic distances.

TABLE S1 Primers used in this study.
