# Supplementary material for: Two P1B-1-ATPases of Amanita strobiliformis With Distinct Properties in Cu/Ag Transport
Source: Front Microbiol. 2018 Apr 23;9:747. doi: 10.3389/fmicb.2018.00747 (PMC5924815; doi:10.3389/fmicb.2018.00747)
Supplement: TABLE S1 — Primers used in this study. [file Table_1.docx]

**Table S1.** Primers used in this study

| Target | Primer name ^a^ | Sequence (5´→3´) |
| --- | --- | --- |
| As*CRD1* | 5rCRD1_1 | CAGTAGCTTCTGGTCGCACTACG |
|  | 5rCRD1_2 | CCAATTCAGCTTCGTGTTCTTGGATCC |
|  | 5rCRD1_3 | TCACTGCCCAACACGGCAGTGC |
|  | 5rCRD1_4 | TACTACCCTGATTATGTCGTCCAGC |
|  | 5rCRD1_5 | GACGCATTGCCGACCCATGC |
|  | eifCRD1_F | CGGTATCGATAAGCTTCTATTGGGATCTCGGTTCTGC |
|  | eifCRD1_R | ATTCGATATCAAGCTTATGGGGACTCTTCACTTCAC |
|  | gifCRD1_F | TAGAACTAGTGGATCCATGGGGACTCTTCACTTCACCG |
|  | gifCRD1_R | TGCTCACCATGGATCCTTGGGATCTCGGTTCTGCACC |
|  | mCRD1_F | GTGTTCGCTAAAACTGGCACGGTGACGG |
|  | mCRD1_R | AGTTTTAGCGAACACTATGATATCTATCTG |
|  | qF-CRD1 | GCCGCAGATGTTGGTATTGC |
|  | qR-CRD1 | ACATCAATGCCCAAGCGAAG |
| As*CRD2* | 5rCRD2_1 | AGCGAGCTGGTCACCACACTCACGC |
|  | 5rCRD2_2 | ATCTGCTCTCCACTGTACC |
|  | 5rCRD2_3 | ACAAGTTCTGTTGGGATGC |
|  | 3rCRD2_1 | GGTGCGGGCGTTGCAATGGTCGGTGACG |
|  | 3rCRD2_2 | TGGTAACATGCGCCGGTCGCAAGCA |
|  | eifCRD2_F | GCTTGATATCGAATTCGCTTCAGACGTTCCGAGTACC |
|  | eifCRD2_R | CGGGCTGCAGGAATTCGACCCATGTCCTTGCTGCTTG |
|  | gifCRD2_F | TAGAACTAGTGGATCCATGTCCTTGCTGCTTGGATCC |
|  | gifCRD2_R | TGCTCACCATGGATCCGACGTTCCGAGTACCCATTTCC |
|  | mCRD2_F | GTGGTGGCCAAGACAGGCACAGTAACTGTG |
|  | mCRD2_ | TGTCTTGGCCACCACAACTCGACGTATATC |
|  | qF-CRD2 | GTCTGTCGCCATCGAAGCTG |
|  | qR_CRD2 | CATTATAGACGCATGCCCAGATC |
| As*TUB-b* | qFtub-b | ACCCTCGTCATGGTCGTTAC |
|  | qRtub-b | TCACACTGAGCGGTGAGAAC |

^a^ Primer description according to the purpose: “5r“, 5´RACE; “3r“: 3´RACE; “eif”, amplification from the whole cDNA pool / the construction of expression vectors; “gif”, the construction of As*CRD*:*GFP* fusions; “m”, site-directed mutagenesis (nucleotide mutations written in lower case); “q”, qRT-PCR analyses. The p416-GPD vector overlaps in “eif” and p416-GFP plasmid overlaps in “gif” primers are italicized. Primers “q” were designed to produce 164 bp, 134 bp and 154 bp amplicons from As*CRD1*, As*CRD2* and As*TUB-b* cDNAs, respectively.
